# Supplementary material for: Economic analysis of open versus laparoscopic versus robot-assisted versus transanal total mesorectal excision in rectal cancer patients: A systematic review
Source: PLoS One. 2023 Jul 28;18(7):e0289090. doi: 10.1371/journal.pone.0289090 (PMC10381040; doi:10.1371/journal.pone.0289090)
Supplement: S2 File — (PDF) [file pone.0289090.s002.pdf]

S2 File: Search syntax

PubMed
("Rectal Neoplasms"[Mesh] OR (Rect*[tiab] AND cancer*[tiab]) OR (Rect*[tiab] AND neoplasm*[tiab]) OR (Rect*[tiab] AND tumo*[tiab]))

AND

((total mesorect*[tiab] AND (excision*[tiab] OR removal*[tiab] OR surger*[tiab])) OR TME[tiab] OR tatme[tiab] OR transanal mesorectal excision*[tiab])

AND

("Laparoscopy"[Mesh] OR Laparo*[tiab] OR "Robotics"[Mesh] OR Robot*[tiab] OR "Transanal Endoscopic Surgery"[Mesh] OR Transanal*[tiab] OR Open[tiab])

Embase
('rectum tumor'/exp OR (Rect*:ab,ti AND cancer*:ab,ti) OR (Rect*:ab,ti AND neoplasm*:ab,ti) OR (Rect*:ab,ti AND tumo*:ab,ti))

AND

((total mesorect*:ab,ti AND (excision*:ab,ti OR removal*:ab,ti OR surger*:ab,ti)) OR TME:ab,ti OR tatme:ab,ti OR transanal mesorectal excision*:ab,ti)

AND

('laparoscopy'/exp OR laparoscop*:ab,ti OR 'robotics'/exp OR robot*:ab,ti OR 'Transanal Endoscopic Surgery'/exp OR Transanal*:ab,ti OR Open*:ab,ti)

Web of Science
("Rectal neoplasms" OR (Rect* AND cancer*) OR (Rect* AND neoplasm*) OR (Rect* AND tumo*))

AND

((total mesorect* AND (excision* OR removal* OR surger*)) OR TME OR tatme OR transanal mesorectal excision*)

AND

("Laparoscopy" OR Laparo* OR "Robotics" OR Robot* OR "Transanal Endoscopic Surgery" OR Transanal* OR Open)

Scopus
("Rectal neoplasms" OR (Rect* AND cancer*) OR (Rect* AND neoplasm*) OR (Rect* AND tumo*))

AND

((total mesorect* AND (excision* OR removal* OR surger*)) OR TME OR tatme OR transanal mesorectal excision*)

AND

("Laparoscopy" OR Laparo* OR "Robotics" OR Robot* OR "Transanal Endoscopic Surgery" OR Transanal* OR Open)

Cochrane Library

ID	Search
#1	MeSH descriptor: [Rectal Neoplasms] explode all trees
#2	(rect*):ti,ab,kw (Word variations have been searched)
#3	(cancer*):ti,ab,kw (Word variations have been searched)
#4	(tumo*):ti,ab,kw (Word variations have been searched)
#5	(total mesorect*):ti,ab,kw (Word variations have been searched)
#6	(excision*):ti,ab,kw (Word variations have been searched)
#7	(removal*):ti,ab,kw (Word variations have been searched)
#8	(surger*):ti,ab,kw (Word variations have been searched)
#9	(TME):ti,ab,kw (Word variations have been searched)
#10	(tatme):ti,ab,kw (Word variations have been searched)
#11	(transanal mesorectal excision*):ti,ab,kw (Word variations have been searched)
#12	MeSH descriptor: [Laparoscopy] explode all trees
#13	(laparo*):ti,ab,kw (Word variations have been searched)
#14	MeSH descriptor: [Robotics] explode all trees
#15	(robot*):ti,ab,kw (Word variations have been searched)
#16	MeSH descriptor: [Transanal Endoscopic Surgery] explode all trees
#17	(transanal*):ti,ab,kw (Word variations have been searched)
#18	(open*):ti,ab,kw (Word variations have been searched)
#19	(#1 OR (#2 AND #3) OR (#2 AND #4)) AND ((#5 AND (#6 OR #7 OR #8)) OR #9 OR #10 OR #11) AND (#12 OR #13 OR #14 OR #15 OR #16 OR #17 OR #18)

Filters and limits

Limits were imposed for date of publication. This review included studies published between January 2000 and December 2023 in the search strategy
