# Supplementary material for: Economic analysis of open versus laparoscopic versus robot-assisted versus transanal total mesorectal excision in rectal cancer patients: A systematic review
Source: PLoS One. 2023 Jul 28;18(7):e0289090. doi: 10.1371/journal.pone.0289090 (PMC10381040; doi:10.1371/journal.pone.0289090)
Supplement: S3 File — (PDF) [file pone.0289090.s003.pdf]

S3 File: Overview of original costs and converted costs

Author	Original costs	Costs after correcting for inflation	Costs after applying purchasing power parity
Baek et al	$62,601.00	$77,791.96	$77,791.96
	$83.915,00	$10,4278.08	$104,278.08
Candido et al	€1,049.22	€1,068.88	$1,634.37
	€1,803.76	€1,837.56	$2,809.72
	€46.00	€46.86	$71.65
	€50.80	€51.75	$79.12
	€39.70	€40.44	$61.83
	€39.50	€40.24	$61.52
	€29.40	€29.00	$44.34
	€39.40	€40.14	$61.37
Elbarmelgi et al	$2,950.00	$2,788.90	$2,788.90
Feng et al	¥40,947.02	¥46,755.86	$11,166.91
Feng et al	$12,395.50	$11,733.61	$11,733.61
	$8,170.90	$7,436.97	$7,436.97
	$9,307.50	$8,471.48	$8,471.48
	$4,266.70	$3,883.46	$3,883.46
	$2,768.20	$2,519.55	$2,519.55
	$3,059.90	$2,785.05	$2,785.05
	$671.90	$611.55	$611.55
	$707.80	$644.22	$644.22
Leung et al	$7,148.00	$10,879.02	$10,879.02
	$9,297.00	$13,336.21	$13,336.21
Morelli et al	€10,242.40	€10,483.64	$16,030.03
	€7,686.80	€7,867.85	$12,030.35
	€1,358.00	€1,389.99	$2,125.36
	€1,195.1	€1,223.25	$1,870.41
	€4,481.20	€4,586.75	$7,013.37
	€3,302.00	€3,379.77	$5,167.84
	€4,380.80	€4,483.98	$6,856.23
	€3,464.40	€3,546.00	$5,422.01
Pai et al	$22,640.00	$25,883.23	$25,883.23
Pan et al	$9,579.97	$8,746.01	$8,746.01
	$13,740.80	$12,544.63	$12,544.63
Park et al	$10,101.30	$11,548.33	$11,548.33
	$12,742.5	$14,567.89	$14,567.89
Ramji et al	$12,558.56	$14,051.07	$11,213.94
	$11,493.56	$12,859.50	$10,262.96
	$18,273.35	$20,445.02	$16,316.85
	$4,339.63	$4,855.37	$3,874.99
	$5,313.59	$5,945.08	$4,744.67
	$11,879.66	$13,291.48	$10,607.72
	$619.66	$693.3	$553.31
	$464.47	$519.67	$414.74
	$583.93	$653.33	$521.41
	$4,392.88	$4,914.95	$3,922.54
	$4,914.33	$5,498.37	$4,388.16
	$4,406.04	$4,929.67	$3,934.29
	$1,015.96	$1,136.7	$907.18
	$1,032.16	$1,154.83	$921.65
	$567.67	$635.13	$506.88
	$42.61	$47.67	$38.04
	$73.15	$81.84	$65.31
	$78.63	$87.97	$70.20
	$501.67	$561.29	$447.95
	$578.64	$647.41	$516.68
	$431.59	$482.88	$385.37
Rouanet et al	€11,172.00	€11,355.48	$15,662.73
	€13,520.00	€13,724.04	$18,929.71
	€4,952.00	€5,033.33	$6,942.52

€4,199.00	€4,267.96	$5,886.84
€1,957.00	€1,989.14	$2,743.64
€3,285.00	€3,338.95	$4,605.44
€713.00	€724.71	$999.60
€870.00	€884.29	$1,219.71
€1,626.00	€1,652.70	$2,279.58
€3,365.00	€3,420.26	$4,717.60
€1,559.00	€1,584.60	$2,185.65
€1,724.00	€1,752.31	$2,416.97
€365.00	€370.99	$511.71
€77.00	€78.26	$107.94
