# Supplementary material for: Economic analysis of open versus laparoscopic versus robot-assisted versus transanal total mesorectal excision in rectal cancer patients: A systematic review
Source: PLoS One. 2023 Jul 28;18(7):e0289090. doi: 10.1371/journal.pone.0289090 (PMC10381040; doi:10.1371/journal.pone.0289090)
Supplement: S4 File — Modified Consensus on Health Economic Criteria (CHEC) checklist tool. Questions used per category and explanatory note are provided. (PDF) [file pone.0289090.s004.pdf]

S4 File: Modified Consensus on Health Economic Criteria (CHEC) checklist

Items	Sub-category questions	Explanation	Answers	%
1	Is the study population clearly described?	Relevant clinical characteristics, entry and eligibility criteria, as well as drop-out during follow-up were reported.	Yes	
		One or more of the above were not reported.	No	
2	Are competing alternatives clearly described?	A clear and specific statement of the techniques to be compared was provided, as well as detailed descriptions of the competing interventions preferably including procedural steps.	Yes	
		No clear and specific statement of the techniques to be compared was provided.	No	
		Study did not compare two or more minimally invasive techniques.	NA	
3	Is a well-defined research question posed in answerable form?	The research question clearly identifies the alternatives being compared and the population for which the comparison is made.	Yes	
		No question/aim/objective has been given. Or the question/aim/objective does not identify the alternatives and population.	No	
4	Is the economic study design appropriate to the stated objective?	The economic study design is a full economic evaluation (comparison of costs and effects of 2 or more interventions) based on primary research (cohort, case-control, randomized controlled trial).	Yes	
		Study design consisting of other than the above or studies using prospectively collected data or previous studies originally designed for assessing other than costs.	No	
5	Is the chosen time horizon appropriate in order to include relevant costs and consequences?	The time horizon is equal for costs and outcomes if these are combined in a ratio. A follow-up period of > 3 months was used.	Yes	
		A follow-up period shorter than 3 months is used.	No	

6	Is the actual perspective chosen appropriate?	Societal perspective or health care provider.<br><br>Other perspective or no perspective given.	Yes<br><br>No
7	Are all important and relevant costs for each alternative identified?	All important and relevant costs (see explanation) were given in relation to the perspective and the research question.<br><br>One or more of the relevant costs are not given (see explanation).<br><br>*Explanation:* importantly, relevant costs were decided depending on study perspective and time horizon. In case of studies investigating only total hospitalization costs these included: theatre costs, non-theatre costs. In case of studies investigating long term costs these included: theatre costs, non-theatre costs, post-discharge costs (readmission, emergency department visits, reinterventions) and indirect costs (housekeeping, medical home care, etc.).	Yes<br><br>No
8	Are all costs measured appropriately in physical units?	Costs were measured in physical units, the instrument by which the costs are measured was valid and clearly stated (e.g. interview, questionnaire, cost-diary).<br><br>The instrument by which the costs are measured was unclear or invalid.	Yes<br><br>No
9	Are costs valued appropriately?	The sources of valuation (i.e. national cost registers, or hospital and factory pricing) were clearly stated for each cost price of every volume parameter and their reference year. The main cost was calculated based on depleted sources, no tariffs were used.<br><br>No clear valuation of each cost price of every volume parameter is given.	Yes<br><br>No
10	Are all important and relevant outcomes for each alternative identified?	All important and relevant outcomes for cost-effectiveness were given in relation to the perspective and the research question.	Yes<br><br>No

		One or more important/relevant outcomes for cost-effectiveness is missing. If all important outcomes are identified but one or more is not assessed, fill in No.	
11	Are all outcomes measured appropriately?	The outcome measurement resulted from the outcome identification and this was straightforward (e.g. if mortality is a main outcome measure this should be taken into account in the analysis). The instrument by which the outcomes are measured was valid and clearly stated.	Yes
		The outcome measurement did not result from the outcome identification. The instrument by which the outcomes are measured was not valid and/or not clearly stated.	No
12	Are outcomes valued appropriately?	The method of outcome valuation was clearly stated. Examples of valuation methods are Discrete Choice Experiments (e.g. Conjoint analysis, Contingent valuation), Direct utility assessment (VAS, TTO, SG, etc.), Indirect utility assessment (HUI, EQ-5D, QWB, etc.), Person trade off, etc.	Yes
		The study was not a primary study assessing costs or cost-effectiveness. Therefore, no method of outcome valuation was stated.	No
13	Is an incremental analysis of costs of alternatives performed?	The incremental analysis examined the additional costs from one intervention over another.	Yes
		No incremental analysis of costs of alternatives was performed or was performed appropriately.	No
		No differences in costs were reported, since this study did not compare two or more minimally invasive techniques.	NA
14	Are all future costs and outcomes discounted appropriately?	Discounting was performed as all costs and outcomes were converted to one single year, based on a motivated discount rate.	Yes

		Discounting was not performed or not performed appropriately.<br><br>Study collected costs of only 1 year.	No<br><br>NA
15	Are all important variables, whose values are uncertain, appropriately subjected to sensitivity analysis?	All variables in the analysis are potential candidates for the sensitivity analysis. Only variables that are certain or which have a minimal impact on the study results (based on the preliminary analysis) can be excluded from the sensitivity analysis. Furthermore, a justification should be given over the range of the variables used in the sensitivity analysis.<br><br>Not all variables in the analysis were subjected to sensitivity analysis.	Yes<br><br>No
16	Do the conclusions follow from the data reported?	Results were interpreted with caution and conclusions were justified by the data.<br><br>Results were interpreted without proper caution, conclusions were not justified by the data or new data not previously provided was presented in the conclusion.	Yes<br><br>No
17	Does the study discuss the generalizability of the results to other settings and patient/client groups?	The study reports explicit about the viewpoint of analysis and indicates how particular costs and outcomes vary by location, setting, patient population, care provider, etc.<br><br>The article did not elaborate on any aspect of generalizability.	Yes<br><br>No
18	Does the article indicate that there is no potential conflict of interest of study researcher(s) and funder(s)?	No potential conflict of interest of study researcher(s) and funder(s) was reported. In case an external agency finances the study, a statement explicitly illustrated who finances the study to guarantee transparency in the relationship between the sponsor and the researcher.<br><br>In case a potential conflict of interest is possible or a declaration was given of ‘competing interest’. If one or both are not stated, fill in no.	Yes<br><br>No

NA, not applicable

Modified Consensus on Health Economic Criteria (CHEC) checklist tool. Questions used per category and explanatory note are provided.
