# Supplementary material for: Economic analysis of open versus laparoscopic versus robot-assisted versus transanal total mesorectal excision in rectal cancer patients: A systematic review
Source: PLoS One. 2023 Jul 28;18(7):e0289090. doi: 10.1371/journal.pone.0289090 (PMC10381040; doi:10.1371/journal.pone.0289090)
Supplement: S5 File — NA, not applicable. The Consensus on Health Economic Criteria (CHEC) checklist is a current research standard for conducting systematic reviews which are based on economic evaluation studies and was used to rate study the quality of all studies included in this review. The results of the CHEC tool indicate risk of bias and applicability concerns. This study used a modified CHEC tool using categorical questions adjusted to study design of the included studies. (PDF) [file pone.0289090.s005.pdf]

S5 File Quality assessment of the included studies using the Consensus on Health Economic Criteria (CHEC) checklist

	Consensus on Health Economic Criteria (CHEC) checklist question																	
Study	1	2	3	4	5	6	7	8	9	10	11	12	13	14	15	16	17	18
Baek et al													NA				NA	
Candido et al																		
Elbarmelgi et al		NA							NA	NA	NA	NA	NA					
Feng et al													NA	NA				
Feng et al																		
Leung et al																		
Morelli et al		NA																
Pai et al		NA																NA
Pan et al																		
Park et al		NA																
Ramji et al		NA											NA					
Rouanet et al		NA																

NA, not applicable

The Consensus on Health Economic Criteria (CHEC) checklist is a current research standard for conducting systematic reviews which are based on economic evaluation studies and was used to rate study the quality of all studies included in this review. The results of the CHEC tool indicate risk of bias and applicability concerns. This study used a modified CHEC tool using categorical questions adjusted to study design of the included studies.
