# Supplementary material for: Economic analysis of open versus laparoscopic versus robot-assisted versus transanal total mesorectal excision in rectal cancer patients: A systematic review
Source: PLoS One. 2023 Jul 28;18(7):e0289090. doi: 10.1371/journal.pone.0289090 (PMC10381040; doi:10.1371/journal.pone.0289090)
Supplement: S1 Table — ASA, American Society of Anesthesiology; BMI, body mass index; L-TME, laparoscopic total mesorectal excision; n, number of patients; O-TME, open total mesorectal excision; R-TME, robotic total mesorectal excision; TaTME, transanal total mesorectal excision; -, not available. (PDF) [file pone.0289090.s006.pdf]

S1 Table: Patients demographics and preoperative characteristics of included studies

First author	Technique	Gender, n (%) Male	Female	Age, mean (years)	BMI, mean	Neoadjuvant therapy, n (%)	Stage, n (%) 0	1	2	3	4	ASA Classification, n (%) I	II	III	IV
Baek et al	L-TME	25 (61)	16 (39)	63.7	26.7	18 (43.9)	3 (7.3)	15 (36.6)	3 (7.3)	19 (46.3)	1 (2.4)	0 (0)	13 (31.7)	26 (63.4)	2 (4.9)
	R-TME	25 (61)	16 (39)	63.6	25.7	33 (80.5)	7 (17.1)	12 (29.3)	4 (9.8)	15 (36.6)	3 (7.3)	0 (0)	18 (43.9)	22 (53.7)	1 (24.4)
Candido et al	L-TME	37 (56.1)	29 (43.9)	64.32	25.40	37 (56)	-	-	-	-	-	10 (15)	44 (67)	11 (17)	1 (1)
	TaTME	63 (73.3)	23 (26.7)	60.50	24.94	56 (65)	-	-	-	-	-	31 (36)	48 (56)	6 (70)	1 (1)
Elbarmelgi et al	TaTME	21 (52.5)	19 (47.5)	50.43	-	40 (100)	0 (0)	4 (10)	17 (42.5)	19 (47.5)	0 (0)	31 (77.5)	9 (22.5)	0 (0)	0 (0)
Feng et al	L-TME	40 (67.8)	19 (32.3)	56.31	22.64	0 (0%	-	-	-	-	-	4 (6.8)	55 (93.2)	0 (0)	0 (0)
Feng et al	L-TME	354 (60.5)	231 (39.5)	60.7	23.5	257 (43.9)	0 (0)	203 (34.7)	200 (34.2)	182 (31.1)	0 (0)	-	-	-	-
	R-TME	356 (60.8)	230 (39.2)	59.1	23.5	254 (43.4)	0 (0)	205 (35.0)	192 (32.8)	189 (32.3)	0 (0)	-	-	-	-
Leung et al	O-TME	114 (57)	86 (43)	66.5	-	55 (27.5)	0 (0)	28 (14)	73 (36.5)	69 (34.5)	30 (15.0)	-	-	-	-
	L-TME	104 (51.2)	99 (48.8)	67.1	-	77 (37.9)	0 (0)	31 (15.3)	72 (35.5)	64 (31.5)	36 (17.7)	-	-	-	-
Morelli et al	Si	24 (60)	16 (40)	69.8	-	18 (45)	-	-	-	-	-	0 (0)	29 (47.5)	21 (52.5)	0 (0)
	Xi	28 (70)	12 (30)	67.0	-	18 (45)	-	-	-	-	-	2 (5)	18 (45)	20 (50)	0 (0)
Pai et al	R-TME	-	-	-	-	-	-	-	-	-	-	-	-	-	-
Pan et al	L-TME	43 (86)	7 (14)	64.0	-	3 (6)	0 (0)	13 (26)	21 (42)	16 (32)	0 (0)	28 (65)	19 (38)	3 (6)	0 (0)
	R-TME	53 (94.6)	3 (5.4)	64.0	-	0 (0)	0 (0)	13 (23.2)	15 (26.8)	28 (50)	0 (0)	36 (64.3)	19 (33.9)	1 (1.8)	0 (0)
Park et al	L-TME	60 (71.4)	24 (28.6)	63.5	22.9	10 (11.9)	0 (0)	22 (26.2)	28 (33.3)	34 (40.5)	0 (0)	51 (60.7)	28 (33.3)	5 (6.0)	0
	R-TME	86 (64.7)	47 (35.3)	59.2	23.1	15 (11.3)	0 (0)	49 (36.8)	36 (27.1)	48 (36.1)	0 (0)	94 (70.7)	31 (23.3)	8 (6.0)	0
Ramji et al	O-TME	15 (57.7)	11 (42.3)	69	27.9	6 (23)	1 (4)	4 (15)	5 (19)	16 (62)	0 (0)	0 (0)	8 (31)	18 (69)	0 (0)
	L-TME	19 (70.4)	8 (29.6)	63.7	27.6	6 (22)	4 (15)	6 (22)	3 (11)	14 (52)	0 (0)	4 (15)	9 (33)	13 (48)	1 (4)
	R-TME	19 (73.1)	7 (26.9)	62.1	27.8	15 (58)	5 (19)	7 (27)	4 (16)	10 (38)	0 (0)	0 (0)	3 (12)	22 (85)	1 (4)
Rouanet et al	L-TME	47 (66)	23 (34)	60	24.4	45 (63)	0 (0)	3 (5)	16 (28)	34 (59)	5 (9)	-	-	-	-
	R-TME	40 (69)	18 (31)	66	25.1	43 (74)	0 (0)	4 (8)	42 (84)	4 (8)	0 (0)	-	-	-	-

ASA, American Society of Anesthesiology; *BMI*, body mass index; *L-TME*, laparoscopic total mesorectal excision; *n*, number of patients; *O-TME*, open total mesorectal excision; *R-TME*, robotic total mesorectal excision; *TaTME*, transanal total mesorectal excision; -, not available
