# Supplementary material for: Economic analysis of open versus laparoscopic versus robot-assisted versus transanal total mesorectal excision in rectal cancer patients: A systematic review
Source: PLoS One. 2023 Jul 28;18(7):e0289090. doi: 10.1371/journal.pone.0289090 (PMC10381040; doi:10.1371/journal.pone.0289090)
Supplement: S2 Table — APR, abdominoperineal resection; CRM+, positive circumferential resection margin; L-TME, laparoscopic total mesorectal excision; LAR, low anterior resection; n, number of patients; NA, not applicable; O-TME, open total mesorectal excision; R-TME, robotic total mesorectal excision; TaTME, transanal total mesorectal excision; -, not available, * Data shows median values, a Other procedures include coloanal anastomosis, intersphincteric resection and Hartmann surgery. (PDF) [file pone.0289090.s007.pdf]

S2 Table: Operative data and postoperative data

		Procedure, n (%)											Clavien-Dindo Classification, n (%)				
First author	Technique	LAR	APR	Other[a]	TaTME, n (%)	Operating time, mean (min)	Stomy formation, n (%)	Conversions, n (%)	CRM+, n (%)	Number of surgeons	Surgeon's experience	Complication rate, n (%)	I	II	III	IV	V
Baek et al	L-TME	33 (80.5)	6 (14.6)	2 (4.9)	NA	315	14 (40)	9 (22)	2 (4.9)	-	-	11 (26.8)	-	-	-	-	-
	R-TME	33 (80.5)	6 (14.6)	2 (4.9)	NA	296	33 (93.5)	3 (7.3)	1 (2.4)	-	-	9 (22)	-	-	-	-	-
Candido et al	L-TME	66 (100)	0 (0)	0 (0)	NA	266	-	2 (3.0)	-	-	-	11 (17)	0 (0)	5 (8)	6 (9)	0 (0)	0 (0)
	TaTME	86 (100)	0 (0)	0 (0)	53 (62)	271	-	0 (0)	-	-	-	17 (20)	4 (5)	9 (10)	4 (5)	0 (0)	0 (0)
Elbarmelgi et al	TaTME	40 (100)	0 (0)	0 (0)	-	179.10	29 (72.5)	1 (2.5)	0 (0)	-	-	3 (7.5)	-	-	-	-	-
Feng et al	L-TME	59 (100)	0 (0)	0 (0)	NA	169.35	0 (0)	3 (5.1)	-	-	3+ years	10 (16.9)	-	-	-	-	-
Feng et al	L-TME	449(76.8)	133(22.7)	3 (0.5)	NA	170.00	117 (26.1)	23 (3.9)	39 (7.2)	13	> 100	135 (23.1)	NA	107(18.3)	24 (4.1)	3 (0.5)	1 (0.2)
	R-TME	486(82.9)	99(16.9)	1 (0.2)	NA	173.00	129 (26.5)	10 (1.7)	22 (4.0)	13	> 100	95 (16.2)	NA	78 (13.3)	15 (2.6)	1 (0.2)	1 (0.2)
Leung et al	O-TME	200 (100)	0 (0)	0 (0)	NA	144.2	-	NA	-	>1	-	45 (22.5)	-	-	-	-	-
	L-TME	203 (100)	0 (0)	0 (0)	NA	189.9	-	47 (23.3)	-	>1	-	40 (19.7)	-	-	-	-	-
Morelli et al	Si	27 (67.5)	7 (17.5)	6 (15)	NA	310.6	-	1 (2.5)	0 (0)	1	-	14 (17.5)	2 (5)	10 (25)	2 (5)	0 (0)	0 (0)
	Xi	29 (72.5)	5 (12.5)	6 (16)	NA	283.1	-	2 (5)	0 (0)	1	-	10 (12.5)	5 (12.5)	4 (10)	1 (2.5)	0 (0)	0 (0)
Pai et al	R-TME	-	-	-	NA	-	-	-	-	-	-	-	-	-	-	-	-
Pan et al	L-TME	38 (76)	12 (24)	(0)	NA	147.5*	27 (54)	0 (0)	1 (1)	1	< 1200	9 (18)	3 (6)	5 (10)	1 (2)	0 (0)	0 (0)
	R-TME	48 (85.7)	8 (14.3)	0 (0)	NA	190*	24 (42.9)	0 (0)	1 (1.8)	1	-	7 (12.5)	0 (0)	4 (7.1)	2 (3.6)	1 (1.8)	0 (0)
Park et al	L-TME	84 (100)	0 (0)	0 (0)	NA	208.8	20 (23.8)	6 (7.1)	6 (7.1)	1	-	24 (28.6)	9 (10.7)	4 (4.8)	9 (10.7)	2 (2.4)	0 (0)
	R-TME	133 (100)	0 (0)	0 (0)	NA	205.7	29 (21.8)	0 (0)	9 (6.8)	1	-	31 (23.3)	14 (10.5)	5 (3.8)	11 (8.3)	1 (7.5)	0 (0)
Ramji et al	O-TME	20 (77)	6 (23)	0 (0)	NA	214	-	NA	1 (3.8)	-	-	13 (50)	5 (19)	2 (8)	4 (15)	2 (8)	0 (0)
	L-TME	16 (60)	11 (40)	0 (0)	NA	240	-	10 (37)	0 (0)	-	-	10 (42)	6 (25)	0 (0)	0 (0)	4 (17)	0 (0)
	R-TME	22 (85)	4 (15)	0 (0)	NA	407	-	3 (12)	0 (0)	-	-	11 (42)	4 (15)	4 (15)	3 (12)	0 (0)	0 (0)
Rouanet et al	L-TME	71 (100)	0 (0)	0 (0)	NA	226*	36 (51)	6 (8)	7 (10)	1	-	-	-	-	-	-	-
	R-TME	58 (100)	0 (0)	0 (0)	NA	233*	34 (59)	3 (5)	6 (10)	1	-	-	-	-	-	-	-

APR, abdominoperineal resection; *CRM+*, positive circumferential resection margin; L-*TME*, laparoscopic total mesorectal excision; *LAR*, low anterior resection; *n*, number of patients; *NA*, not applicable; *O-TME*, open total mesorectal excision; *R-TME*, robotic total mesorectal excision; *TaTME*, transanal total mesorectal excision; -, not available

* Data shows median values

[a] Other procedures include coloanal anastomosis, intersphincteric resection and Hartmann surgery
